# Repurposing a microfluidic formulation device for automated DNA construction

**DOI:** 10.1371/journal.pone.0242157

**Published:** 2020-11-11

**Authors:** Garima Goyal, Nick Elsbree, Michael Fero, Nathan J. Hillson, Gregory Linshiz

**Affiliations:** 1 Technology Division, DOE Joint BioEnergy Institute, Emeryville, California, United States of America; 2 Biological Systems & Engineering Division, Lawrence Berkeley National Laboratory, Berkeley, California, United States of America; 3 TeselaGen Biotechnology, San Francisco, California, United States of America; MAHSA University, Malaysia, MALAYSIA

## Abstract

Microfluidic applications have expanded greatly over the past decade. For the most part, however, each microfluidics platform is developed with a specific task in mind, rather than as a general-purpose device with a wide-range of functionality. Here, we show how a microfluidic system, originally developed to investigate protein phase behavior, can be modified and repurposed for another application, namely DNA construction. We added new programable controllers to direct the flow of reagents across the chip. We designed the assembly of a combinatorial Golden Gate DNA library using TeselaGen DESIGN software and used the repurposed microfluidics platform to assemble the designed library from off-chip prepared DNA assembly pieces. Further experiments verified the sequences and function of the on-chip assembled DNA constructs.

## Introduction

In recent years, researchers in different fields have developed a diversity of microfluidics technologies. Microfluidics systems have been applied to parallel gene synthesis; PCR; DNA purification, separation, and sequencing; Next Generation Sequencing sample preparation; DNA sizing by single molecule detection; DNA hybridization arrays; drug discovery; pathogen identification; cellular analysis; biochemical clinical assays; protein arrays; and biosensors [1–8]. Microfluidic technologies offer high-throughput operations, precise fluid control, and low reagent and power energy consumption [9–12]. It remains challenging, however, to develop a single flexible microfluidic device that may be used for multiple applications, since each application may have different (and often conflicting) operational requirements.

One possible alternative, pursued herein, is to start with an existing microfluidic device designed for a specific function, and attempt to repurpose it (potentially with some modifications) for an alternative application. Here, we begin with a microfluidic formulation device, which was previously designed to perform functions such as the combinatorial mixing of 16 buffers and 16 precipitation agents with a purified protein sample. This chip showed the possibility and practicality to access a vast number of chemical conditions and to accurately dispense and mix fluids on the picoliter scale [2].

**Fig 1:**
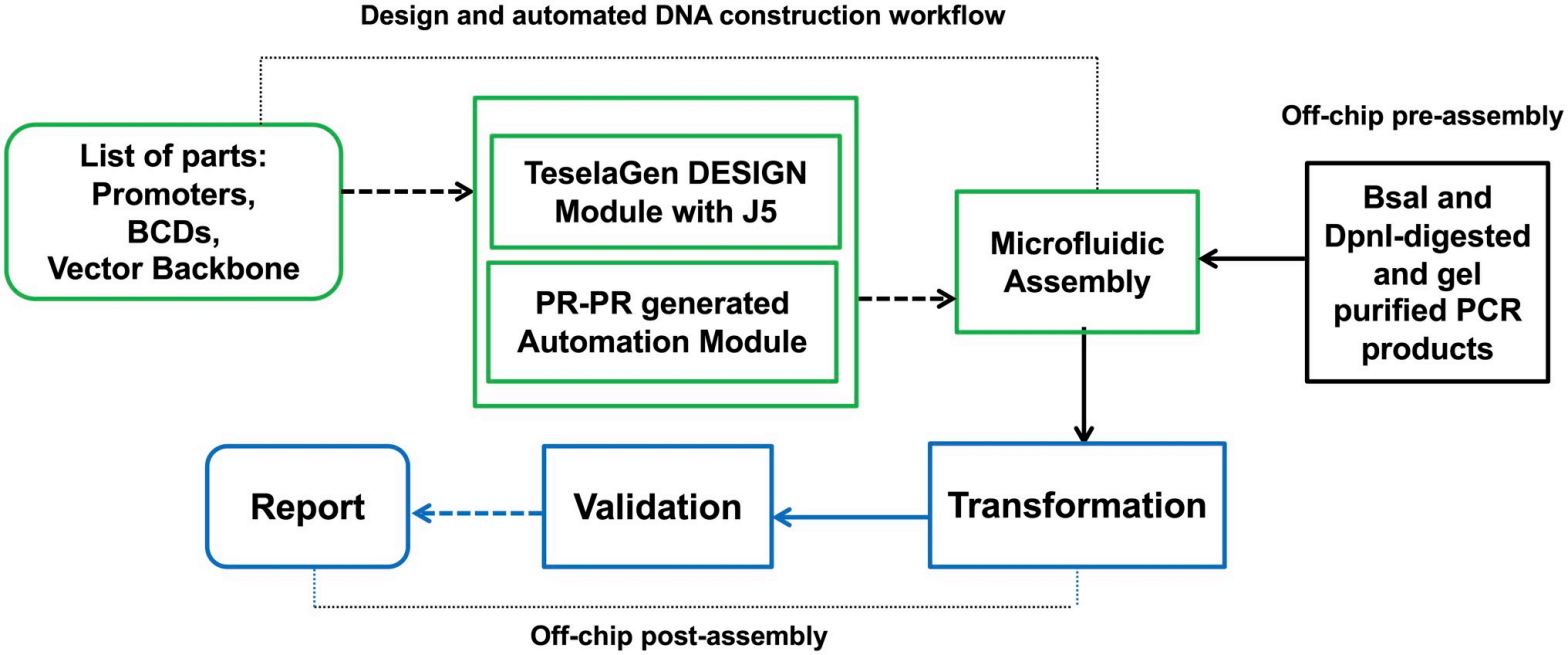
DNA design and construction workflow on and off a microfluidic device. User requests are sent from the TeselaGen DESIGN module to j5 to generate a scar-less combinatorial Golden Gate DNA assembly protocol. The automation module allocates resources, and initiates construction. Gel-purified, BsaI and DpnI-digested PCR products are generated off-chip according to the protocol, and then supplied to and then assembled together on the microfluidic chip. Assembled DNA (plasmids) are transformed into a host system of interest (*E*. *coli*) off-chip. The assembled DNA can then be sequence validated, and resulting reports generated.

Previously, we developed PR-PR [13–15], a high-level user-friendly programming language for laboratory automation, which supports microfluidics platforms. The new work reported here further integrates manually generated PR-PR scripts with microfluidics to maneuver the controller of the repurposed microfluidic device to construct of DNA using a j5 [16]-designed Golden Gate assembly method [17–19] protocol. TeselaGen’s DESIGN module was used to specify the combinatorial DNA library design, and interface with j5 to design Golden Gate DNA assembly protocol. Gel-purified, BsaI and DpnI-digested DNA assembly pieces were prepared (per j5 protocol) off-chip, and then assembled on-chip. Thereafter, assembly reaction mixtures were transformed off-chip into *E*. *coli* and sequence validated and functionally tested. The DNA design and construction workflow on a microfluidic device is shown in Fig 1.

## Results and discussion

### Integration of PR-PR with a microfluidic controller

**Fig 2:**
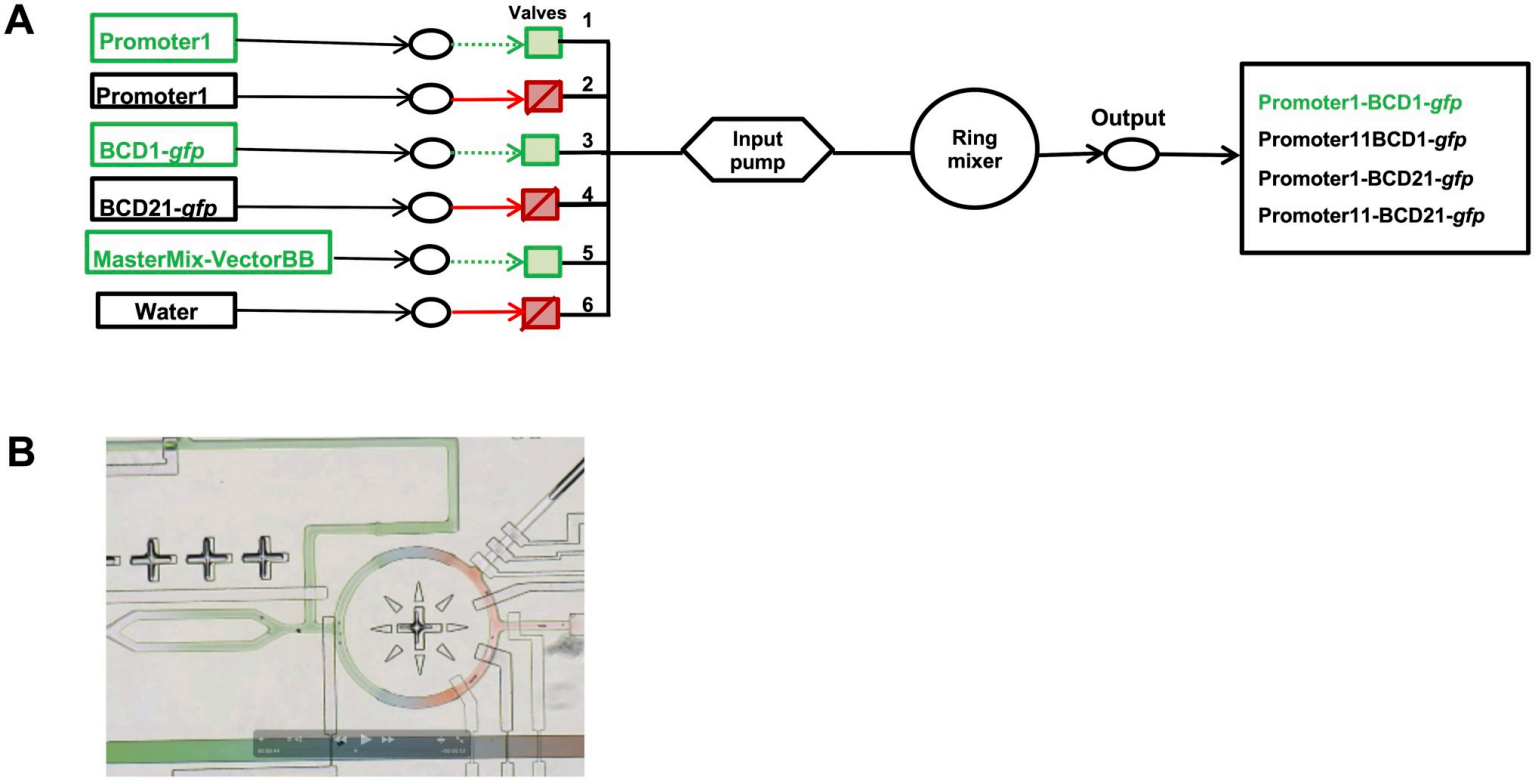
**A**. Schematic of the basic flow structure of the microfluidic chip. Six input valves are controlled by an input pump. A ring mixer is used for mixing the reagents and the output well is for collecting the mixed reagents. E.g.—valves 1, 3, and 5 are open to allow Promoter1, BCD1-*gfp* and MasterMix-VectorBB reagents to pass through valves. These reagents are mixed in ring mixer and finally get collected in the output well. **B**. Still video frame of liquid flow and combinatorial mixing on the microfluidic chip.

To control the repurposed microfluidic device, we integrated PR-PR [14, 15] with a microfluidic controller operated by MATLAB. The MATLAB code for the controller was closely borrowed from K Brower et al. [20]. Manually generated PR-PR scripts consist of three parts: 1) the location of each component, 2) the description of the DNA assembly Golden Gate protocol, and 3) the execution of the protocol. The locations of input and output components were pre-determined on the chip. Valves 1–6, as shown in Fig 2, were controlled by the PR-PR scripts. For example, the script Promoter1BCD1_GFP.pr (provided in S1 Promoter) controls the input and output components by opening valves 1, 3, and 5 and closing the others so that reagents from the opened valves can flow through the ring mixer for incubation to achieve on-chip DNA assembly and this assembly reaction is then sent on to the output valve where the DNA assembly is collected for off-chip transformation. Each transfer step in the protocol is defined by Source (input reagents), Destination (output), and Volume (of each reagent). Since different liquid types vary in viscosities and for precise liquid control, we have defined delay times in the script to compensate for these variations in viscosity. Similar procedures are followed in the other PR-PR scripts. S1 Fig illustrates the microfluidic test rig used in this study.

### DNA assembly

To demonstrate the application of the repurposed microfluidic device to DNA construction, we chose to pursue the combinatorial Golden Gate assembly of the library shown in Fig 2A. To design the Golden Gate DNA assembly protocol, we used TeselaGen’s DESIGN module (https://teselagen.com/design-build-platform/) to specify the library, as shown in Fig 2B. The TeselaGen software then calls j5 [16] to generate the Golden Gate DNA assembly protocol [16–19, 21, 22] DNA parts were prepared following the j5-designed protocol. The Golden Gate DNA assembly protocol by j5 was then translated manually to a PR-PR script for execution of the DNA assembly reactions on the microfluidic device. The j5-designed protocol was also used off-chip (both manual and conventional liquid-handling laboratory automation devices) to guide the PCR, DpnI and BsaI digest, and gel-purification of the DNA assembly pieces.

### Microfluidic reagent flow

**Fig 3:**
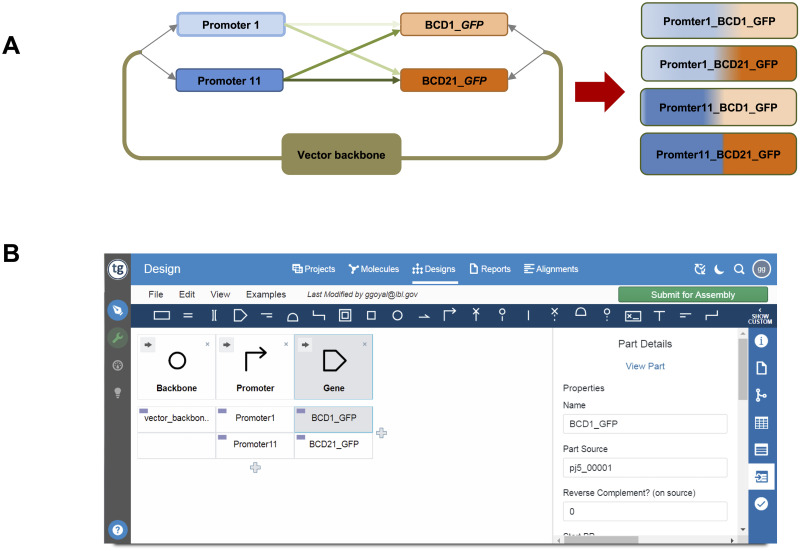
**A**. Schematic of combinatorial assembly of pre-made DNA assembly pieces using the Golden Gate method on the microfluidic chip. **B**. A screenshot of the TeselaGen DESIGN module (classic view)–design of the combinatorial library: a vector backbone (first column), two promoters (second column), and two (bicistronic) ribosomal binding sites followed by *gfp* (third column).

Our repurposed microfluidic chip was designed specifically for formulation and so has the requisite hiearical valving design need for combinatorial DNA assembly. Additionally, the chip features a metering scheme that allows for sequential injection of precise sample aliquots from a single microfluidic channel into an array of reaction chambers through a positive displacement crossinjection (PCI) junction. The PCI junction is formed by the combination of a three-valve peristaltic pump and a four-port crossinjection junction with integrated valves on each port [2]. Each cycle of the peristaltic pump injects a well defined volume of sample (≈80 pl), determined by the dead volume under the middle valve of the peristaltic pump. Reagent flow from six input wells into the chip is controlled by individually controlled valves. Valves can be opened and closed to selectively mix specific reagents together. Reagents flow sequentially into the ring mixer where they combine. The resulting combined mixture then flows to and can be collected from the output well. For our DNA construction application, five different reagents: Promoter 1, Promoter 11, BCD1-*gfp*, BCD21-*gfp*, and the vector backbone (mixed with ligase); were dispensed into the input wells. To assemble the construct Promoter1-BCD1-*gfp* (for example), valves 1, 3, and 5 were open and valves 2, 4, and 6 were closed, as shown in Fig 3A. To avoid cross-contamination between assembly reactions, the input pump and channels were washed with water by opening valve 6 and closing the others. We integrated PR-PR with MATLAB [20] to control the chip [14, 15]. A still video frame of the ring mixer in action is shown in Fig 3B. PR-PR scripts (provided in Supplementary Information) were used to operate the microfluidic device.

### Combinatorial DNA library design using the TeselaGen DESIGN module

We selected to pursue a subset of 4 constructs (of 308) from a previously published library (combinations of 22 constitutive promoters with 14 bicistronic devices followed by *gfp* [23]). We used the TeselaGen DESIGN module to visually design the 4 variant combinatorial plasmid DNA library, with a common vector backbone, two promoter variants, and two bicistronic device (BCD) variants coupled with a *gfp* gene (Fig 3). The TeselaGen DESIGN module calls j5 to design the combinatorial Golden Gate DNA assembly protocol to build the plasmid library. We opted for a modified Golden Gate approach, in which the DNA fragments to be assembled are BsaI digested and gel-purified prior to ligation [17, 19].

### Assembled DNA constructs sequence and functional validation

**Fig 4:**
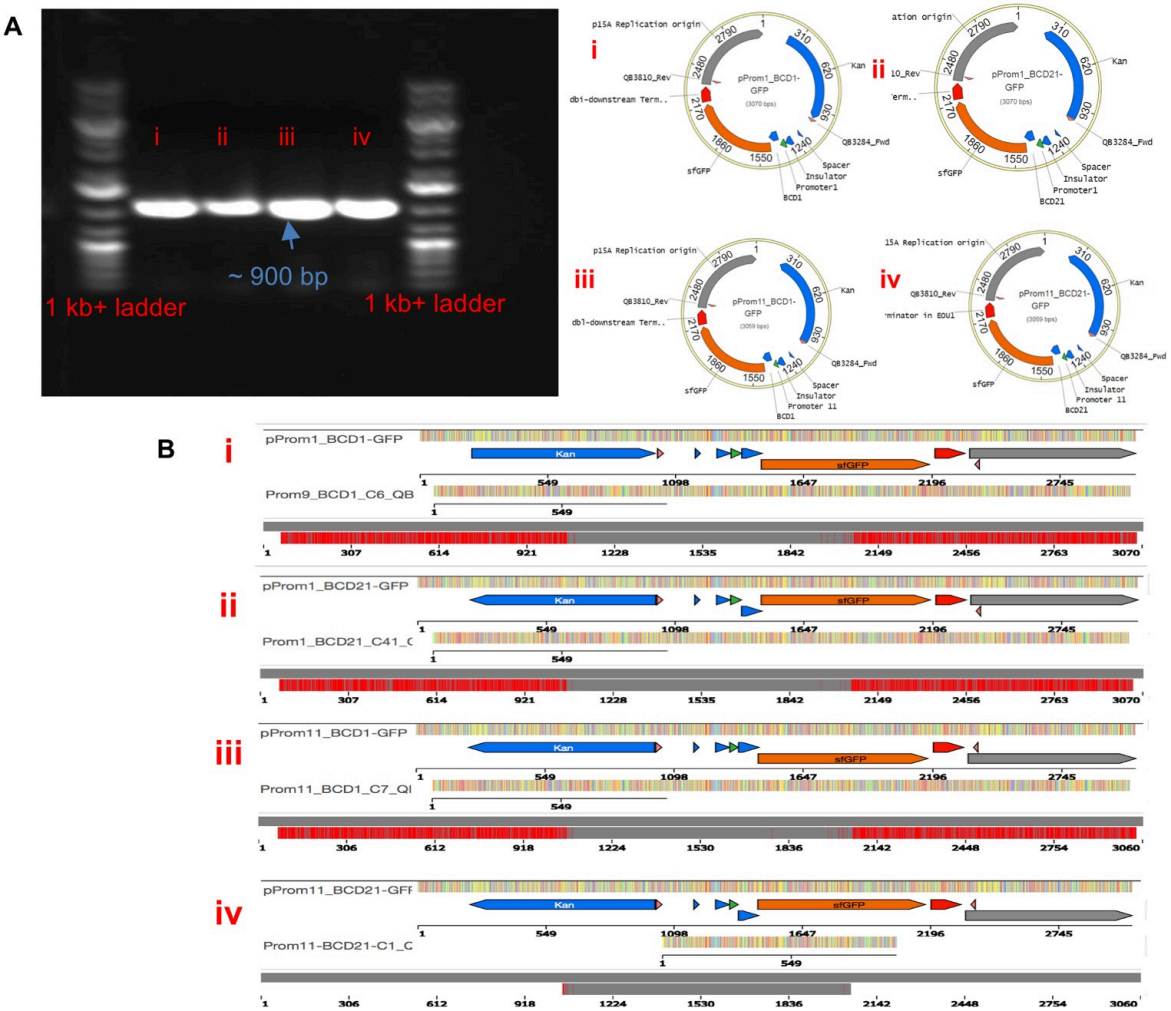
**A**. Colony PCR gel image: colony PCR was performed using oligos QB3284_Fwd and QB3810_Rev, generating a fragment of approximately 900 bp in size and plasmid maps for all constructs. **B**. Sanger sequence validation traces for the 900 bp fragment with no mutations for the constructs (i) Prom1BCD1, (ii) Prom1BCD21, (iii) Prom11BCD1, and (iv) Prom11BCD21.

**Fig 5:**
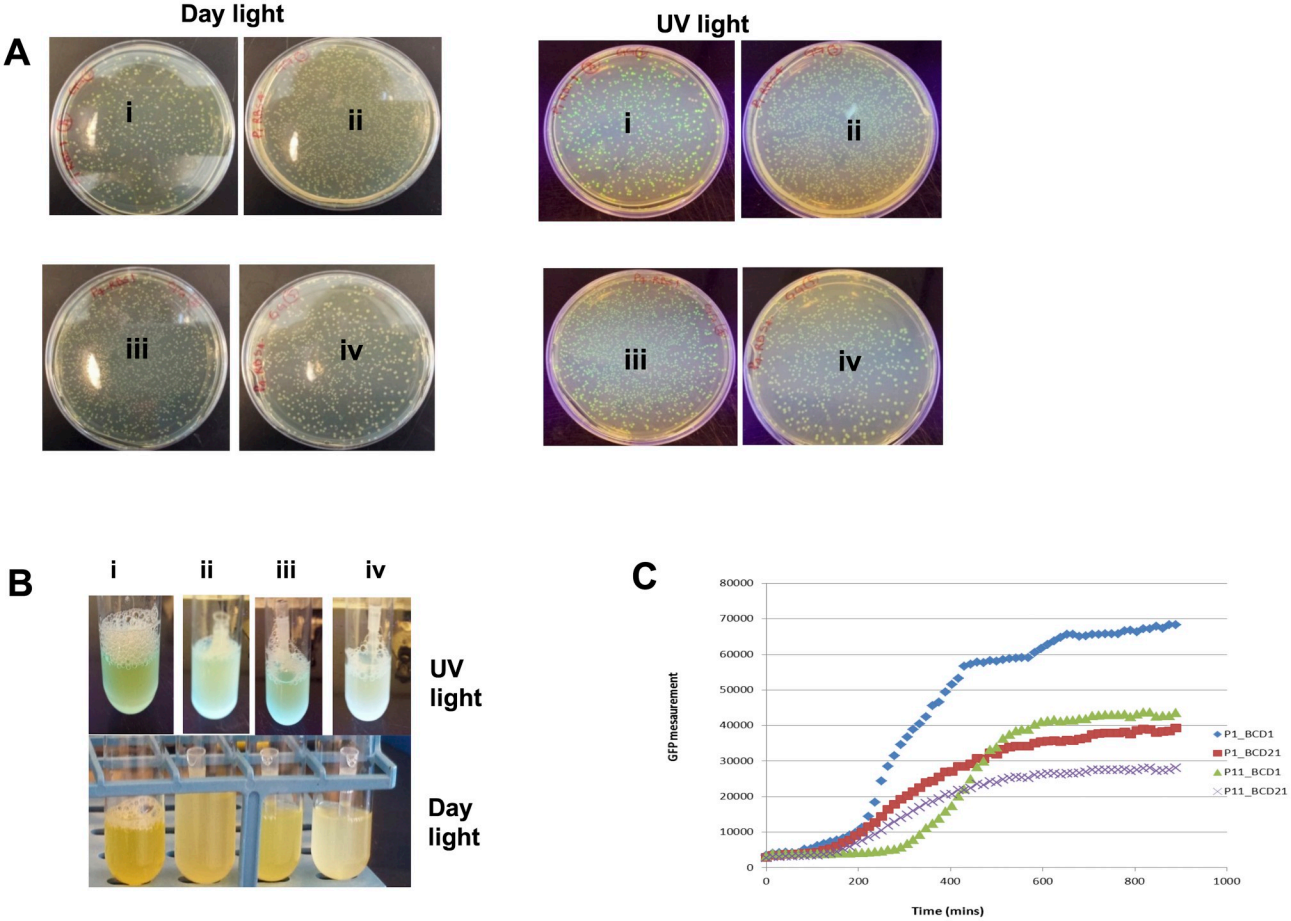
**A**. Agar (LB + Kanamycin (40μg/mL)) plates after heat-shock transformation, showing hundreds of colonies under normal light and UV light (overlapping excitation wavelength of GFP). (i) Prom1_BCD1-*gfp*, (ii) Prom1_BCD21-*gfp*, (iii) Prom11_BCD1-*gfp*, (iv) Prom11_BCD21-*gfp*. **B**. Liquid cultures of fluorescing GFP constructs under normal light and UV light. (i) Prom1_BCD1-*gfp*, (ii) Prom1_BCD21-*gfp*, (iii) Prom11_BCD1-*gfp*, (iv) Prom11_BCD21-*gfp* **C**. Micro-plate reader assay for GFP fluorescence measurement. As expected, Prom1_BCD1-*gfp* is exhibiting the highest fluorescence.

We transformed off-chip each of the resulting 4 DNA assembly reactions into *E*. *coli* DH10B, and grew the transformed cells on selective LB plates overnight. One transformant colony from each assembly reaction was picked for further screening. Colony PCR and Sanger sequencing validated the expected sequences of all 4 cloned plasmids (all combinatorial variants), for the plasmid sequence region spanning the spacer, insulator, promoter, BCD, *gfp*, and terminator as shown Fig 4. Microplate reader assays further confirmed the expected results of variations in GFP fluorescence. Fig 5 shows that construct Prom1_BCD1-*gfp*, which contains strongest promoter and BCD, exhibited highest GFP fluorescence, followed by Prom11_BCD1-*gfp*, Prom1_BCD21-*gfp*, and Prom11_BCD21-*gfp*. A slight delay of ~80 mins to kickstart the fluorescence was observed for construct Promoter11_BCD1_GFP. As observed in Mutalik et al [22] where these constructs were originally reported, varying strengths of promoters and engineered bicistronic ribosomal binding sites provide a greater control over transcription and translation initiation in *E*. *coli* e.g. in case of Promoter11_BCD1_GFP, a small change in the strength of promoter could result in a quantitatively measurable change in the translation initiation which explains the delay in time for fluorescence to kick-start. We only used a subset of 4 constructs from a library of 308 constructs to illustrate the DNA construction capabilities on a pre-existing microfluidic chip. To understand the engineering of expression cassettes, more details can be found in the Mutalik et al publication [22].

These results importantly demonstrate that the repurposed microfluidic device, in combination with controller programed by PR-PR operated by MATLAB [20], can be successfully used for DNA construction. Further, we have compared the on-chip generated DNA assembly of the four DNA constructs used in this study with the manually generated DNA assembly reported in previously published reports [12, 14] for the same constructs. We observed comparable DNA assembly and transformation efficiency results for both the platforms. Emittance of green color due to GFP in liquid culture and bacterial colonies qualitatively confirmed the correct assembly of all the three DNA pieces. All the colonies screened for both the platforms yielded 100% sequence verified DNA constructs. We attribute, since the on-chip and off-chip assemblies result in the same DNA sequences (confirmed via Sanger Sequencing), any difference in GFP fluorescence intensities observed to biological replicate variations within *E*. *coli* and not to the platform used for DNA construction.

More testing is needed to further establish the efficiency and repeatability of the microfluidic device. We do not anticipate any new challenges will emerge while applying the device to other DNA assembly strategies (such as Gibson Assembly), considering that the pump and valves controlled by PR-PR script on a microfluidic device will remain the same.

## Methods

All DNA oligos were purchased from Integrated DNA Technologies (Coralville, IA). DNA assembly reagents and restriction enzymes were purchased from New England Biolabs (Ipswich, MA). Unless otherwise specified, all chemicals, solvents, and media components were purchased and used without modification from Sigma-Aldrich (St. Louis, MO), Fisher Scientific (Pittsburgh, PA), or VWR (West Chester, PA).

### PCR amplification

pFAB4876, and pFAB4932, extracted from *E*. *coli* using Spin Miniprep kits (Qiagen; Valencia, CA), served as DNA templates for the PCR amplification of promoter fragments Promoter1, and Promoter11, respectively. Similarly, pFAB4876, and pFAB4883 served as DNA templates for the PCR amplification of the two BCD variant fragments BCD1_*gfp*, and BCD21_*gfp*, respectively. pFAB4876 served as the DNA template for the PCR amplification of the vector backbone. Primers (IDT; Coralville, Iowa) used for the PCR amplifications are listed in S1 Table. Primers MS_02148_(Backbone_p4001)_forward and MS_02149_(Backbone_p4001)_reverse were used for the amplification of the vector backbone; primers MS_02150_(P1)_forward and MS_02151_(P1)_reverse, and MS_02154_(P2)_forward and MS_02161_(P11)_reverse were used for the amplification of fragments Promoter1, and Promoter11, respectively; and primers MS_02152_(BCD1-GFP)_forward and MS_02153_(BCD1-GFP)_reverse were used for the amplification of the two BCD variant fragments. 50 μL PCR reactions consisted of 2.5 μL (2.5 μM) of each forward and reverse primer, 1 μL template, 1 μL dNTPs (10 mM), 0.5 μL of high-fidelity Phusion polymerase (BioRad; Hercules, CA), 10 μL of 5x high-fidelity Phusion buffer, and 32.5 μL deionized water. Two PCR reactions (100 μL total) were performed for each fragment amplified. The following PCR thermocycling conditions were used: denaturation at 98°C for 30 sec, 35 cycles of denaturation at 98°C for 10 sec, annealing at 68°C for 30 sec, and elongation at 72°C for 30 sec, and a final extension at 72°C for 10 min. The complete list of oligos used for DNA amplification for plasmid construction and colony PCR is provided in S1 Table. Plasmids and strains (S2 Table. used in this study, along with annotated DNA sequences, have been deposited in the public instance of the Joint BioEnergy Institute Registry [22] (https://public-registry.jbei.org/folders/427) and are physically available from the authors and/or addgene (http://www.addgene.org) upon reasonable request.

### DpnI digest and purification

Following PCR amplification, residual (methylated) DNA template in each PCR reaction was DpnI digested at 37°C for 1 h. Each 110 μL digest reaction consisted of 95 μL PCR product, 11 μL 10x Fast Digest buffer, 1.5 μL Fast Digest DpnI (Thermo Fisher Scientific; Waltham, MA), and 2.5 μL deionized water. DpnI was inactivated at 80°C for 5 min, and DNA purification of each DpnI reaction was conducted with a PCR purification kit (Qiagen; Valencia, CA) according to the manufacturer’s protocol, each purified sample eluted with 50 μL of elution buffer.

### BsaI digest and gel purification

Following DpnI digest and purification, 70 μL BsaI digestion reactions consisting of 50 μL purified DpnI reaction, 7 μL NEB4 buffer, 0.7 μL BSA, 5 μL BsaI, and 7.3 μL deionized water, were performed overnight at 37°C. BsaI was deactivated at 65 °C for 20 min. Digested samples were run on a 0.8% agarose gel followed by gel purification kit (Qiagen; Valencia, CA) according to manufacturer’s protocol. Golden Gate protocols were derived from the previously reported articles, in which the DNA fragments to be assembled are BsaI digested and gel purified prior to ligase DNA assembly [17, 18].

### Microfluidic DNA assembly

The Golden Gate DNA assembly scripts (see Supplementary information) were compiled by PR-PR [14, 15] for execution on the microfluidic device. Each of the 4 combinatorial DNA ligation reactions (30 min at room temperature) yielding pProm1_BCD1-GFP … pProm11_BCD21-GFP contained 8 μL ligation reaction master mix, 1 μL BsaI-digested promoter fragment, and 1 μL BsaI-digested BCD variant fragment. Each 8 μL ligation reaction master mix included 1 μL BsaI-digested vector backbone, 1 μL of T4 ligase enzyme (Thermo Scientific; Waltham, MA), 1 μL of T4 ligase buffer, and 5 μL deionized water. Given the limited number of input and output wells available on the microfluidic device, we executed the microfluidic DNA assembly protocol separately for each DNA assembly.

### Transformation

7 μL of each DNA assembly reaction was mixed with 50 μL *E*. *coli* DH10β competent cells [24] (one assembly reaction per aliquot of competent cells) and incubated for 5 min on ice, and then heat shocked at 42°C for 90 s. The cells were returned to ice for 2 min, and there after 100 μL SOC media was added and the culture was incubated at 37°C for 1 h. 35 μL of each transformed culture was plated on LB agar supplemented with 40 μg/mL kanamycin and then incubated at 37°C overnight. Culture tubes containing 10 mL LB media supplemented with 40 μg/mL kanamycin were inoculated with transformants (one picked colony per tube), and placed at 37°C at 900 rpm in a Multitron shaker (Inforys-HT; Basel, Switzerland) overnight (~16 hrs). Transformant glycerol stocks were made from each overnight growth culture and stored at -80°C.

### Qualitative and quantitative analysis

Liquid cultures and bacterial colonies of all variants were qualitatively analyzed by inspection under the day light and UV light. The Promoter1_BCD1-*gfp* variant with the strongest promoter and RBS emits the brightest green color as can be seen in Fig 5A and 5B. Quantitative analysis was performed by micro-plate reader. A Tecan F200 Pro (Tecan, Männedorf Switzerland) microplate reader was used to quantitatively measure GFP fluorescence over 12 hours. Variants confirmed by Sanger sequencing and colony PCR were grown overnight in 5 ml of LB–kanamycin media. 2 uL from each culture was mixed with 148 uL (starting OD_600_ approx. 0.1) of LB-kanamycin media in a 96 well black clear bottom micro-plate reader plate. DI water and LB media were used as blanks. Measurement of GFP fluorescence was performed using the Tecan microplate reader with excitation/emission at 485 nm/ 530 nm. Captured data were transferred directly into an Excel file for direct data analysis. As can be seen in Fig 5C, strongest promoter and RBS combination is producing the highest GFP emissions.

### Colony PCR and Sanger sequencing

Colony PCR was performed for each transformant using 2 μL overnight growth culture as template with primers (10 μM, IDT, S1 Table) and 25 μL PCR reaction for colony PCR consisted of 1.25 μL (2.5 μM) of each forward and reverse primer, 2 μL template, 0.5 μL dNTPs (10 mM), 0.5 μL of Taq polymerase (New England Biolabs), 2.5 μL of 10x Taq reaction buffer, and 17 μL deionized water. The following PCR thermocycling conditions were used: denaturation at 95°C for 30 sec, 35 cycles of denaturation at 95°C for 10 sec, annealing at 60°C for 30 sec, and elongation at 72°C for 30 sec, and a final extension at 72°C for 5 min. Each transformant colony was also submitted for Sanger sequencing (Quintara Bio; Albany, CA) with primers QB3284_Fwd and QB3810_Rev (IDT, S1 Table).

## Conclusion

In this study, we adapted a microfluidic formulation device, originally developed for the investigation of protein phase behavior, for DNA construction applications. We demonstrated on-chip, TeselaGen-designed, Golden Gate DNA assembly of DNA assembly pieces, and sequence and functionally validated the resulting DNA constructs off-chip via colony PCR, Sanger sequencing, and micro-plate reader assays. We anticipate that this same chip could be further adapted to other applications as well, such as DNA construction via assembly methods other than Golden Gate, or performing enzymatic assays. Remaining technical objectives would include, for example, the further standardization of chips, controllers, and high-level programming languages and user-interfaces across microfluidic platforms, as well as the further development of programable general purpose microfluidics platforms.

## Supporting information

S1 Fig The microfluidics test rig is illustrated schematically (top) and is shown in the photo (bottom). (TIF)

S1 Table Primers designed by TeselaGen’s DESIGN module/j5 for Golden Gate DNA assembly. (DOCX)

S2 Table Strains and plasmids. Plasmids and strains used in this study, along with annotated DNA sequences, have been deposited in the public instance of the Joint BioEnergy Institute Registry [20] (https://public-registry.jbei.org/folders/427) and are physically available from the authors and/or addgene (http://www.addgene.org) upon reasonable request.
(DOCX)

S1 Raw image (TIF)

S1 Promoter (PR)

S2 Promoter (PR)

S3 Promoter (PR)

S4 Promoter (PR)
