# Supplementary figures and images for: Repurposing a microfluidic formulation device for automated DNA construction
Source: PLoS One. 2020 Nov 11;15(11):e0242157. doi: 10.1371/journal.pone.0242157 (PMC7657503; doi:10.1371/journal.pone.0242157)

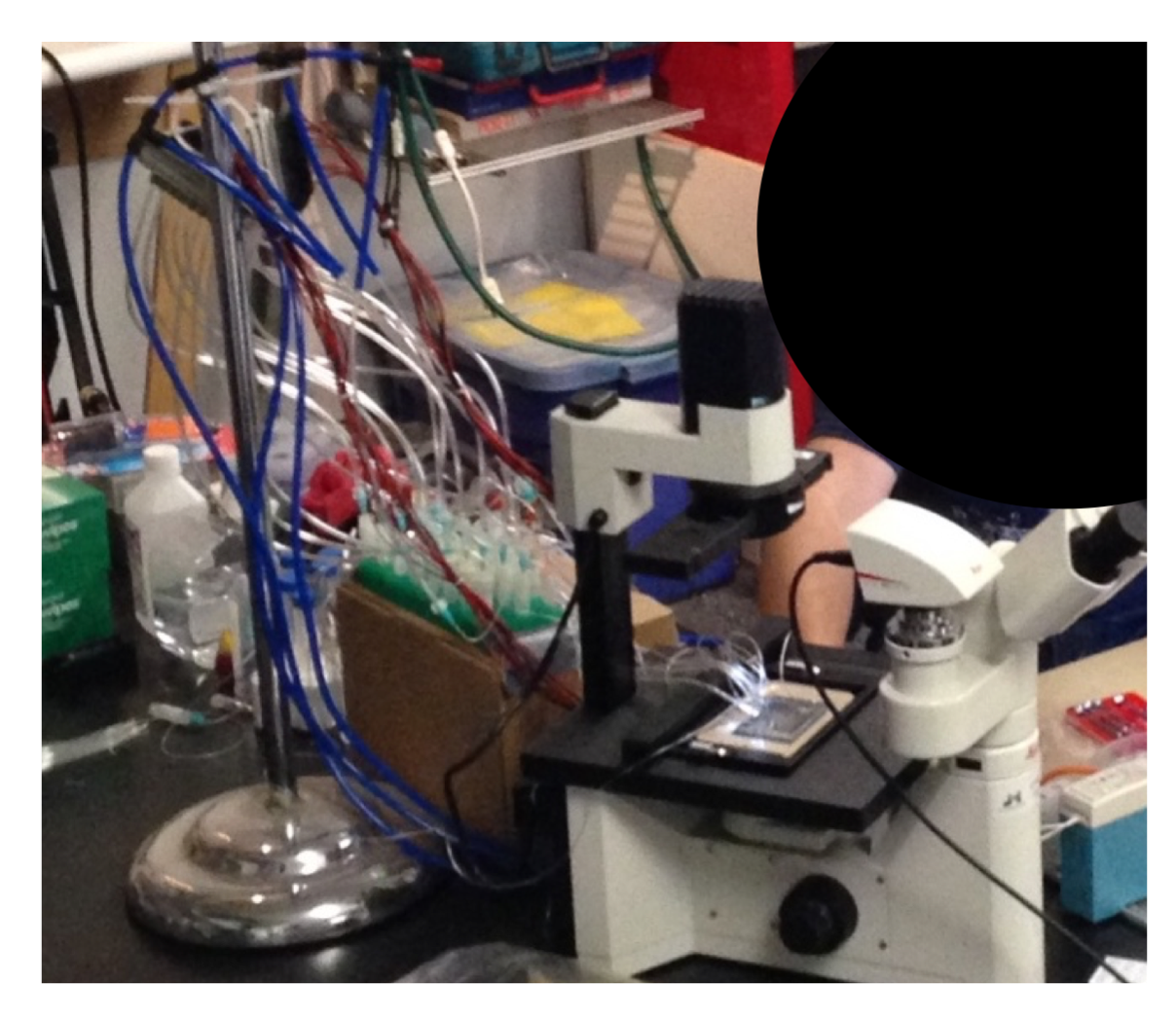

Supplement: S1 Fig — (TIF) [file pone.0242157.s001.tif]

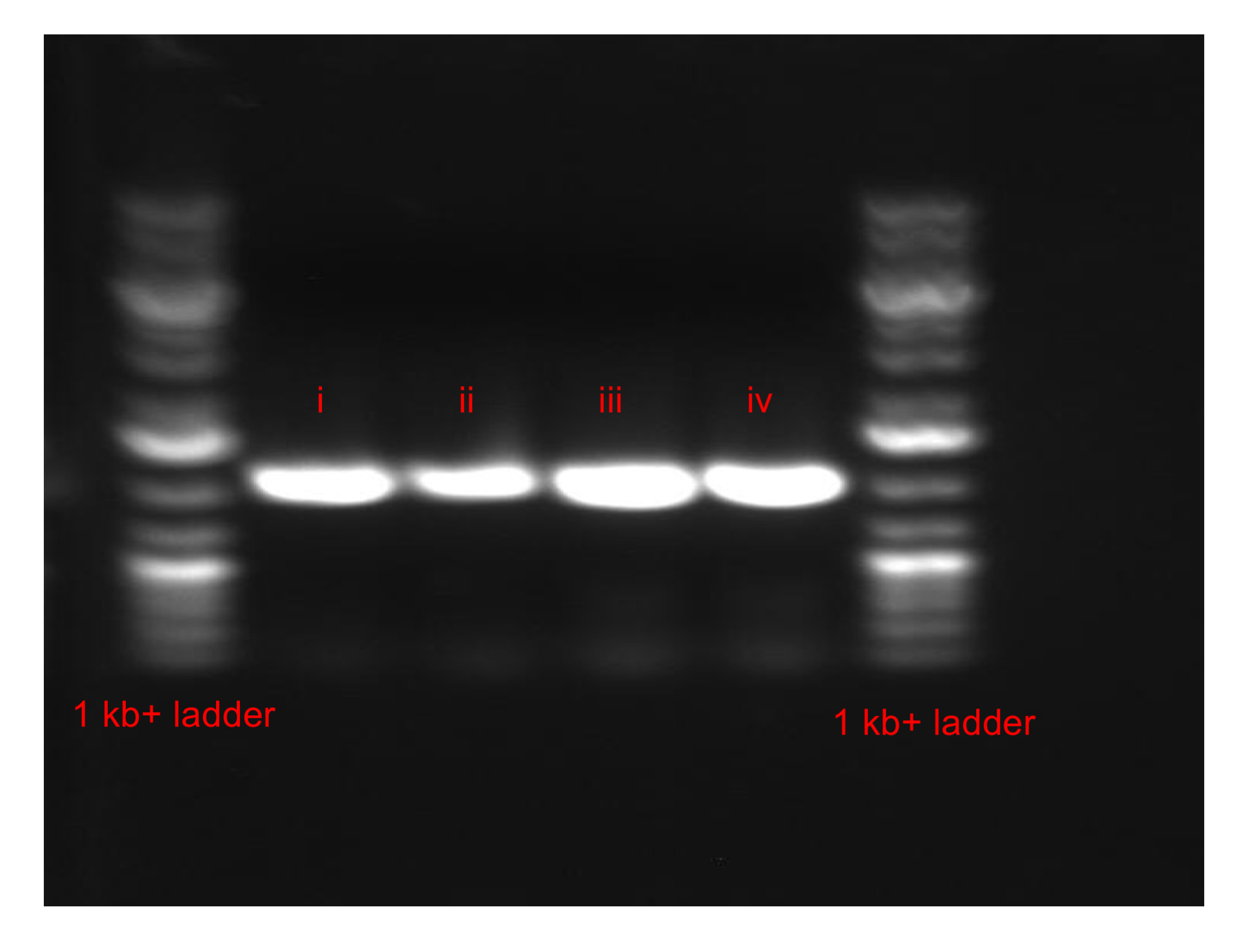

Supplement: S1 Raw image — (TIF) [file pone.0242157.s004.tif]
