# Supplementary material for: Repurposing a microfluidic formulation device for automated DNA construction
Source: PLoS One. 2020 Nov 11;15(11):e0242157. doi: 10.1371/journal.pone.0242157 (PMC7657503; doi:10.1371/journal.pone.0242157)
Supplement: S1 Table — (DOCX) [file pone.0242157.s002.docx]

**S1 Table.** Primers designed by TeselaGen’s DESIGN Module/j5 for Golden Gate DNA Assembly.

| **Oligo Name** | **Length** | **Tm** | **Tm (3' only)** | **Sequence** |
| --- | --- | --- | --- | --- |
| MS_02148_(Backbone_p4001)_forward | 38 | 67.37 | 60.04 | CACACCAGGTCTCAATAAGGATCGGTTGTCGAGTAAGG |
| MS_02149_(Backbone_p4001)_reverse | 46 | 65.44 | 60.49 | CACACCAGGTCTCATCTTACTTACTTAAGATCTTTTGAATTCGACG |
| MS_02150_(P1)_forward | 43 | 65.93 | 60.15 | CACACCAGGTCTCAAAGAGTATACGTATATCGGCTAATAACGT |
| MS_02151_(P1)_reverse | 43 | 67.06 | 59.43 | CACACCAGGTCTCACCTCCACACATTATACCTATAGGTTAGAC |
| MS_02152_(BCD1-GFP)_forward | 39 | 69.29 | 62.26 | CACACCAGGTCTCAGAGGGCCCAAGTTCACTTAAAAAGG |
| MS_02153_(BCD1-GFP)_reverse | 38 | 66.74 | 60.87 | CACACCAGGTCTCATTATTTGTAGAGCTCATCCATGCC |
| MS_02154_(P2)_forward | 50 | 66.25 | 62.84 | CACACCAGGTCTCAAAGAGTATACGTATATCGGCTAATAACGTATTAAGG |
| MS_02161_(P11)_reverse | 36 | 74.47 | 60.11 | CACACCAGGTCTCACCTCCACAAACACTAAGAGCCG |
| QB3284_Fwd | 24 | 56.5 | 56.5 | CGATCCTCATCCTGTCTCTTGATC |
| QB3810_Rev | 19 | 57.9 | 57.9 | CGAGCGTAGCGAGTCAGTG |
