# Supplementary material for: Repurposing a microfluidic formulation device for automated DNA construction
Source: PLoS One. 2020 Nov 11;15(11):e0242157. doi: 10.1371/journal.pone.0242157 (PMC7657503; doi:10.1371/journal.pone.0242157)
Supplement: S2 Table — Plasmids and strains used in this study, along with annotated DNA sequences, have been deposited in the public instance of the Joint BioEnergy Institute Registry [20] (https://public-registry.jbei.org/folders/427) and are physically available from the authors and/or addgene (http://www.addgene.org) upon reasonable request. (DOCX) [file pone.0242157.s003.docx]

**S2 Table.** Strains and plasmids.

Plasmids and strains used in this study, along with annotated DNA sequences, have been deposited in the public instance of the Joint BioEnergy Institute Registry[^20^](#_ENREF_20) (https://public-registry.jbei.org/folders/427) and are physically available from the authors and/or addgene (http://www.addgene.org) upon reasonable request.

| **Plasmid Name** | **Plasmid Description** | **Plasmid JPUB Number** | **Strain Description** | **Strain JPUB Number** | **Ref** |
| --- | --- | --- | --- | --- | --- |
| pFAB4876 | Template for amplifying Promoter1 and BCD1-*gfp* | JPUB_004958 | *E. coli* strain carrying plasmid pFAB4876 | JPUB_004978 | 21 |
| pFAB4932 | Template for amplifying Promoter11 | JPUB_001399 | *E. coli* strain carrying plasmid pFAB4932 | JPUB_004990 | 21 |
| pFAB4883 | Template for amplifying BCD21-*gfp* | JPUB_001396 | *E. coli* strain carrying plasmid pFAB4883 | JPUB_004981 | 21 |
| pProm1_BCD1- *gfp* | P15A ori; Promoter1, BCD1-*gfp* | JPUB_001384 | pProm1_BCD1- *gfp* transformed into *E. coli* DH10B | JPUB_004978 | 13 |
| pProm1_BCD21- *gfp* | P15A ori; Promoter1, BCD21-*gfp* | JPUB_001381 | pProm1_BCD21- *gfp* transformed into *E. coli* DH10B | JPUB_004981 | 13 |
| pProm11_BCD1- *gfp* | P15A ori; Promoter11, BCD1-*gfp* | JPUB_001372 | pProm11_BCD1-*gfp* transformed into *E. coli* DH10B | JPUB_004990 | 13 |
| pProm11_BCD21- *gfp* | P15A ori; Promoter11, BCD21-*gfp* | JPUB_001415 | pProm11_BCD21- *gfp* transformed into *E. coli* DH10B | JPUB_004993 | 13 |
